# The effect of temperature constraints on the treatment of tumors using focused ultrasound-induced acoustic streaming

**DOI:** 10.1038/s41598-024-83782-w

**Published:** 2025-01-02

**Authors:** Sebastian E. N. Price, Magnus Aa. Gjennestad, Signe Kjelstrup, Rune Hansen

**Affiliations:** 1 Porelab and Department of Chemistry, The Norwegian University of Science and Technology NTNU, 7491 Trondheim, Norway; 2 SINTEF Energy Research, Torgarden, PO Box 4761, 7465 Trondheim, Norway; 3 SINTEF, Department of Health Research and Department of Circulation and Medical Imaging, The Norwegian University of Science and Technology NTNU, 7491 Trondheim, Norway

**Keywords:** Biophysics, Cancer, Diseases, Medical research, Mathematics and computing

## Abstract

The transport of drugs into tumor cells near the center of the tumor is known to be severely hindered due to the high interstitial pressure and poor vascularization. The aim of this work is to investigate the possibility to induce acoustic streaming in a tumor. Two tumor cases (breast and abdomen) are simulated to find the acoustic streaming and temperature rise, while varying the focused ultrasound transducer radius, frequency, and power for a constant duty cycle (1%). In the absence of perfusion, the simulated rise in temperature, despite the low duty cycle, never reaches a steady state and is fitted to a logarithmic equation, enabling predictions of the temperature for long treatment times. Higher frequencies and larger probe radii are found to result in shorter treatment times relative to the temperature rise, at the cost of a smaller treated area. Results from the simulations indicate that it may be possible to achieve reasonable acoustic streaming values in tumor without the temperature exceeding 50 °C. Treatment times for streaming a distance of 50 μm in the breast case are shown to range from less than one and a half hour to 93 h, depending on the probe settings.

## Introduction

In the field of tumor treatment, a new and promising approach has emerged involving encapsulation of medication within nanoparticles (NPs), wherein the loaded NPs are introduced to the bloodstream via injection. Interestingly, when these NPs are combined with focused ultrasound (FUS), improved distribution has been shown^1^. Various mechanisms have been proposed to explain this phenomenon^2,3^, including *acoustic streaming*^4,5^. However, a recent study employing single-particle tracking of NPs within an agarose hydrogel in combination with FUS, suggested minimal acoustic streaming effects^6^.

Acoustic streaming is the net, time-averaged movement of a fluid induced by sound waves. In porous tissue, a source of streaming is the acoustic radiation force^7,8^, which is a result of ultrasound (US) dissipation. Increasing the FUS intensity will lead to increased dissipation and therefore increased streaming. However, US dissipation also increases the temperature in the tumor and the near surroundings. At high enough temperatures the proteins in the tumor may denauturate, which may impede the movement of NPs through tissue and be detrimental to the treatment. There is thus a limit to how large the temperatures can become during FUS exposure for physiological reasons. The temperature therefore limits the amount of acoustic streaming displacement that can be induced during a given treatment time. In this work, we aim to explore the potential for inducing discernible acoustic streaming effects within a tumor without exceeding safe temperature limits. The aim is to find optimal transducer parameters to achieve this.

Recent work has advanced the understanding of acoustic streaming phenomena within tissue. Raghavan^9^ formulated equations for acoustic streaming within soft porous media. Subsequently, he utilized his equations to compute acoustic streaming and compared his calculations with prior studies on ultrasound-enhanced convective transport. In some cases, the model predictions were found to agree with the experimental results. Boroumand et al.^10^ employed the model of Raghavan^9^ in their investigations of a brain tissue-mimicking gel. Their comparative analysis revealed that the model predicted experimental outcomes with little deviation. Manor^7^ and Price et al.^8^ presented also equations for acoustic streaming in porous media. These differed, however, from those of Raghavan^9^ in that they did not include a forcing function arising from mass conservation. Price et al.^8^ compared the prediction of their model to experimental results of^11^ and obtained reasonable agreement. Løvmo et al.^12^, using an equation similar to Manor^7^, predicted marginal acoustic streaming effects in their collagen gel experiments on the transport of NPs.

Extensive work has already been made in the direction of explaining a temperature increase due to FUS^13–18^. Huang et al.^16^ conducted comprehensive investigations, successfully measuring and simulating the temperature elevation that results from applying FUS on an agar-based graphite. Notably, they accounted for the viscous effects stemming from the insertion of the thermocouple; a persistent challenge in such experiments. Gray et al.^17,18^ modeled the rise in temperature from ultrasound-mediated hyperthermia for targeted drug delivery. Their methodology involved mapping different tissue types from imaging data into their model. Results were mirroring measured results. Less attention has been devoted to the connection between acoustic streaming and heating. Yuan^19^, building upon Raghavan’s^9^ equation, introduced the concept of “squeezing interstitial fluid via transfer of ultrasound momentum” to augment the model. This refined equation, coupled with the Pennes’ bioheat equation^20^, enabled the simulation of fluid flow and temperature rise in hypothetical FUS treatment scenarios within soft porous matter. Furthermore, Wessapan and Rattanadecho^21^ adopted a Brinkman-extended Darcy model^22^ to compute acoustic streaming during high intensity focused ultrasound (HIFU) treatments in tissue. Employing Pennes’ bioheat equation^20^ for temperature rise, they explored how acoustic streaming influenced temperature elevation during tissue treatment with HIFU. Notably, their utilization of high permeability values led to relatively high acoustic streaming estimates for being in a tissue.

Currently, there is a lack of studies on the possibility to induce acoustic streaming in tumors and also on how the attainable streaming effects are limited by an allowable temperature increase. We shall therefore simulate acoustic streaming for two tumor cases, in breast and abdomen, and calculate the resulting increase in temperature. The time needed to induce a given displacement of fluid will be assessed. Our choice of case studies is motivated by their commonness^23^ and their relatively FUS-accessible position in the body. We shall estimate the temperature increase and treatment time that are necessary to induce a certain displacement for typical ultrasound parameters. The propagation and attenuation of the non-linear ultrasound fields will be modelled using the wide angle Khohklov–Zablotkaya–Kuznetsov (WAKZK) equation^24^ and the acoustic streaming is subsequently calculated using equations from^8^. The temperature evolution during initial sonication is calculated using Pennes’ bioheat equation^20^ and extrapolated forward in time using an empirical equation.

The remaining of this article is structured as follows. In “Equations for acoustic streaming and rise in temperature” section, we present the models we are using. This is followed by “Methods” section, where we explain the two tumor cases and how we numerically calculate the properties. Lastly, in “Results and discussion” section, we show our results and discuss them.

## Equations for acoustic streaming and rise in temperature

We present the models used to estimate acoustic streaming, the resulting rise in temperature and the ultrasound propagation.

### Acoustic streaming

In previous work^8^, we derived equations describing acoustic streaming in soft porous media driven by focused ultrasound, starting from the well-established perturbation approach^25,26^. We applied this approach to the conservation of mass and momentum balance equations, expanding the density $\rho$, pressure *p* and the velocity ${{\mathbf{v}}}$ and retaining only second-order terms, and took their time average. This resulted in well-known acoustic streaming equations valid on the pore scale. These equations were then volume-averaged, following a similar procedure as Whitaker^27^, which gave the time- and spatially averaged volumetric flux due to acoustic streaming,

1 $$\begin{aligned} {\mathbf{q}}&= - K \left\{ \nabla \bar{p}_2 - \bar{{\mathbf{F}}}_2 \right\} . \end{aligned}$$

Herein, *K* is the hydraulic conductivity of the porous medium, $\bar{p}_2$ is the time-averaged second-order pressure and $\bar{{\mathbf{F}}}_2$ is the acoustic radiation force. Conservation of mass requires that ${\mathbf{q}}$ is divergence free, i.e.,

2 $$\begin{aligned} \nabla \cdot {{\mathbf{q}}} = 0. \end{aligned}$$

Combining (1) and (2) gives the Poisson-like equation

3 $$\begin{aligned} \nabla \cdot \left\{ K (\nabla \bar{p}_2 - \bar{{\mathbf{F}}}_2 ) \right\}&= 0, \end{aligned}$$

which may be solved for $\bar{p}_2$ by applying appropriate boundary conditions. The volumetric streaming flux may subsequently be computed from Eq. (1). The velocity in the propagation direction is calculated from the volumetric flux as

4 $$\begin{aligned} v_z=\text {DC}\frac{q_z}{\phi } \end{aligned}$$

where DC denotes the duty cycle of the probe and $\phi$ denotes the porosity of the medium. The time $t_{\Delta z}$ it takes for the fluid to flow a specified distance $\Delta z$ in the propagation direction is

5 $$\begin{aligned} t_{\Delta z}=\frac{\Delta z}{v_z}. \end{aligned}$$

### Temperature

In order to calculate the rise in temperature due to FUS, we utilize Pennes’ bioheat equation^20^,

6 $$\begin{aligned} \rho _0 C_p\frac{\partial T}{\partial t}=\nabla \cdot \kappa \nabla T + Q, \end{aligned}$$

where *T* is the temperature, $C_p$ is the heat capacity of tissue, *Q* a heat source and $\kappa$ is the thermal conductivity of the tissue. The heat source *Q* will here be the heat generated from dissipation of the FUS.

In (6), the perfusion term, which will act as a heat sink and limit the rise in temperature, has been neglected. The reason for neglecting it is that we observed a wide variation, spanning several orders of magnitude, in reported perfusion values in tumors, depending on factors such as tumor type, stage of development, and location within the tumor^28^. The effect is that our simulations will be conservative, i.e. a worst-case scenario, for the temperature increase.

Bacon and Shaw^15^ derived an analytical solution for a special case of (6) with absence of perfusion in the homogeneous material and a constant axisymmetric Gaussian beam. The rise in temperature of the material was then

7 $$\begin{aligned} \Delta T(t)=\frac{\alpha I_0 w^2}{2\kappa }\ln \left( 1+4\frac{\kappa t}{w^2\rho _0 C_p}\right) . \end{aligned}$$

Here $I_0$ is the intensity at the focus, $\alpha$ is the acoustic attenuation coefficient and *w* is the beam radius for the Gaussian beam.

### Ultrasound propagation

In order to solve the above equations for temperature and acoustic streaming, we need to calculate the absorbed heat *Q* and the acoustic radiation force $\bar{{\mathbf{F}}}_2$. To do this, we employ the generalized one-way Westerwelt equation for the propagating pressure field,

8 $$\begin{aligned} \frac{\partial ^2 p}{\partial t^2}-c_0^2\nabla ^2p+2c_0\frac{\partial \{\alpha (\omega )*p(\omega )\}}{\partial t}=\frac{\beta }{\rho _0c_0^2}\frac{\partial ^2 p^2}{\partial t^2}, \end{aligned}$$

where $\omega$ is the angular frequency, $\beta$ is the coefficient of nonlinearity defined as $\beta =1+B/(2A)$, where *B*/*A* is the parameter of nonlinearity, $c_0$ is the speed of sound and $*$ signifies the convolution operation. The propagation pressure field may be expressed as a sum of harmonic contributions

9 $$\begin{aligned} p=P_0\sum _j\left( A_n\exp (i\omega n(z/c_0-t))+A_n^*\exp ( -i\omega n(z/c_0-t))\right) . \end{aligned}$$

Here $A_n$ is the complex amplitude function, which is independent of time *t*, of harmonic component *n*, $P_0$ is the pressure amplitude at the source and *i* is imaginary unit. It is assumed that the real part of the attenuation follows the power law

10 $$\begin{aligned} \text {Re}[\alpha (f)]=\alpha _0\left( \frac{f}{f_0}\right) ^l, \end{aligned}$$

where $f_0={1}\,\hbox {MHz}$, $\alpha _0$ is the attenuation at $f_0$ and *l* is the exponent, which has a value between 0 and 2. The differential form of the Kramers-Kronig relations is used to calculate the phase velocity dispersion from the attenuation. The generalized one-way frequency-domain Westerwelt equation can be written on dimensionless form using $\zeta =z/a$, where *z* is the propagation coordinate and *a* is the probe radius,

11 $$\begin{aligned} \frac{\partial A_n}{\partial \zeta }+[ikan(1-\sqrt{1+X_n})+a\alpha _n]A_n=-\frac{i\beta P_0kan}{2\rho _0c_0^2}\sum _mA_m(A_{n-m}+2A_{m-n}^*). \end{aligned}$$

Here *k* is the wavenumber, $X_n=\nabla _\perp ^2/(kan)^2$ with $\nabla _\perp ^2$ being the transverse Laplacian, acting perpendicular to the propagation direction *z* and superscript $*$ signifies taking the complex conjugate. By using the wide-angle parabolic approximation on the generalized one-way frequency-domain Westerwelt equation (11), neglecting scattering and reflection, we end up with the WAKZK equation^24^. We use this equation to calculate the propagating pressure field. The heat source *Q* may subsequently be calculated as

12 $$\begin{aligned} Q=\frac{P_0^2}{\rho _0c_0}\left( \sum _n\text {Re}(\alpha _n)|A_n|^2\right) . \end{aligned}$$

In order to calculate the acoustic radiation force we use the plane wave approximation,

13 $$\begin{aligned} \bar{{\mathbf{F}}}_2=-\frac{\nabla \cdot{\mathbf{I}}}{c_0}=\frac{Q}{c_0}\hat{\mathbf {z}}, \end{aligned}$$

where $\mathbf {I}$ is the intensity. This is a similar approach to that of Solovchuk et al.^29^.

## Methods

We present the tumor cases under study; i.e. the breast and the abdomen cases, along with numerical methods in use.

In every simulated tumor treatment, we first calculated the ultrasound propagation pressure field. Then, the temperature of the tumor and the corresponding acoustic streaming were calculated.

From the acoustic streaming velocity, the time needed to induce 10 μm of displacement of liquid was computed. The distance 10 μm has been reported to be one of the shorter common distances from blood vessels to cells^30^ and is therefore taken as a benchmark extravasation distance for drugs.

The maximum temperature was determined in the simulation domain during and after the required treatment time. The maximum temperature was compared to the threshold value of 50 °C, the typical minimum temperature used for thermal ablation^31^. At this temperature and above, rapid cell death occurs due to coagulation and protein denaturation.

### Tumor treatment

The breast and abdomen cases are common cases^23^ that are well-positioned for ultrasound exposure, as there are no bone obstructing the ultrasound waves. In all simulation cases, a 1% duty cycle (DC) and a pulse repetition frequency (PRF) of 1 Hz were employed for different configurations (frequency, amplitude and probe radius) of the spherical-segment ultrasound probe. The simulation domains were in all cases made up of regions that are either water, breast tissue, abdomen tissue or tumor tissue. Common water and tumor as well as specific parameters for breast and abdomen are presented in Table 1. The choice of variables is motivated below.

Given the inherent variability in abdomen tissue composition across patients, we selected values within the range typically encountered in the literature^32,33^. The same variability is true for the breast tissue. We drew upon data from Duck^32^ for the speed of sound, coefficient of nonlinearity, attenuation, and its coefficient. Furthermore, we derived the heat capacity, density, and thermal conductivity from Camilleri et al.^34^, assuming a breast tissue composition of 50% breast fat and 50% fibroglandular tissue. Recognizing the inherent heterogeneity of tumors, we opted to assign identical properties to both the breast and abdomen tumors. The speed of sound and coefficient of nonlinearity were set based on the findings of Sehgal et al.^35^, while the attenuation and its coefficient were informed by Barrere et al.^36^. Thermal properties were sourced from Duck^32^.

**Table 1:** Parameters used for the different media in our simulations.

Property	Unit	Symbol	Water	Breast	Abdomen	Tumor
Speed of sound	$\hbox {ms}^{-1}$	$c_0$	1480	1510	1500	1500
Attenuation	Np $\hbox {m}^{-1}$	$\alpha _0$	0.025	8.6	5.0	10.0
Exponent in attenuation power law	–	*l*	2.0	1.5	1.1	1.4
Coefficient of nonlinearity	–	$\beta =1+\frac{B}{2A}$	3.500	5.815	4.000	4.000
Hydraulic conductivity	${\hbox {m}^{2}}\,{\hbox {Pa}^{-1}}\,{\hbox {s}^{-1}}$	*K*	N/A	$5.0 \times 10^{-13}$	$5.0 \times 10^{-13}$	$5.0 \times 10^{-13}$
Density	$\hbox {kg}\,\hbox{m}^{-3}$	$\rho _0$	1000	999	1050	1060
Heat capacity	${\hbox {Jkg}^{-1}}\,{\hbox {K}^{-1}}$	$C_p$	4180	2590	3000	3700
Thermal conductivity	${\hbox {Wm}^{-1}}\,\hbox {K}^{-1}$	$\kappa$	0.600	0.262	0.500	0.500

From previous work^8^, we expect that a critical tumor property for acoustic streaming is the hydraulic conductivity. In experimental data, we noted significant variability in this parameter, ranging from 10^−15^ to 10^−12^ m^2^ Pa^−1^ s^−1^ , with typical values around $10^{-14}$ to 10^−13^ m^2^ Pa^−1^ s^−1^^37–42^. In this study, we use a value of 5 × 10^−13^ m^2^ Pa^−1^ s^−1^, which is in the upper range of typical values. This choice means that we simulate a relatively favorable scenario regarding hydraulic conductivity’s influence on acoustic streaming. The porosity of a tumor is highly variable ranging from 0.15 to 0.6^43,44^, we therefore decided to use a value somewhat in the middle, of 0.4.

#### Breast case

**Fig. 1:**
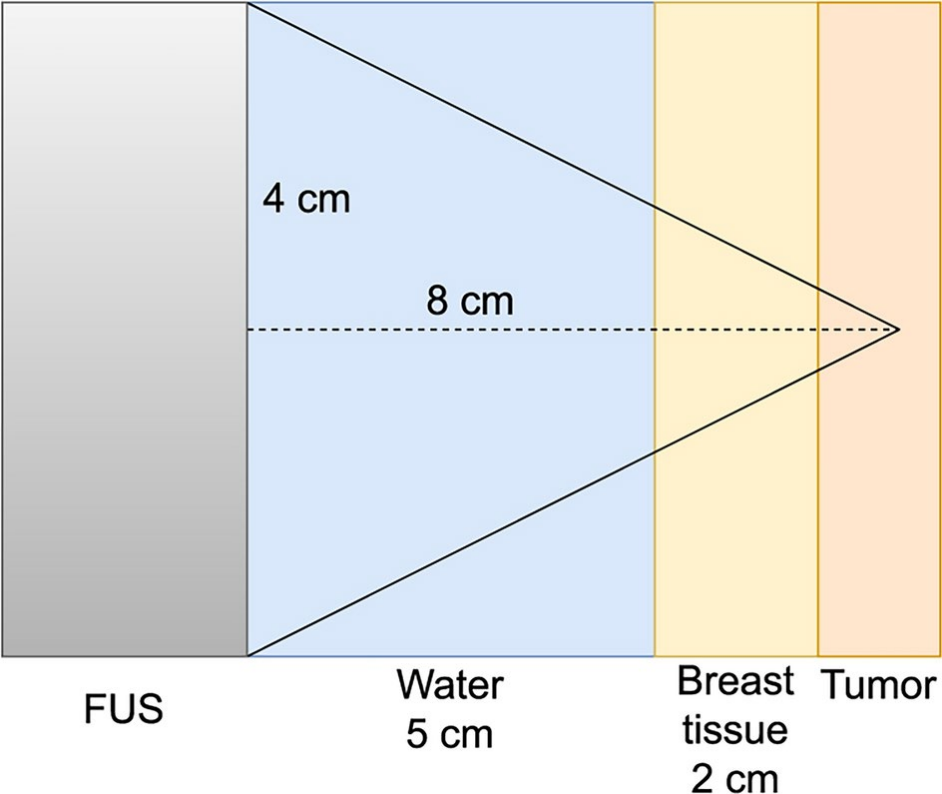
An illustration of the breast base case. The tumor tissue is shown as the orange area, the yellow area shows the breast tissue and the blue area shows the water bath. The FUS probe is to the left-hand side indicated as gray area. In the base case simulation, the probe radius was ${4.0}\,\hbox {cm}$ and the focusing depth was ${8.0}\,\hbox {cm}$.

We explored the treatment of a cancerous tumor in breast using the system as depicted in Fig. 1. The ultrasound probe was positioned ${5}\,\hbox {cm}$ away from the breast, with a water bath in between. The tumor, in this setup, is located ${2}\,\hbox {cm}$ into the breast, separated from the water bath by intervening breast tissue. Our baseline ultrasound probe parameters for this case included a frequency of ${1}\,\hbox {MHz}$, a radius of ${4}\,\hbox {cm}$, a focusing diameter of ${8}\,\hbox {cm}$, and an acoustic power of ${100}\,\hbox {W}$. In addition probe radii of ${3}\,\hbox {cm}$ and ${4}\,\hbox {cm}$, frequencies of ${0.5}\,\hbox {MHz}$ and ${2}\,\hbox {MHz}$ and power of ${50}\,\hbox {W}$ and ${200}\,\hbox {W}$ were also tested.

#### Abdomen case

**Fig. 2:**
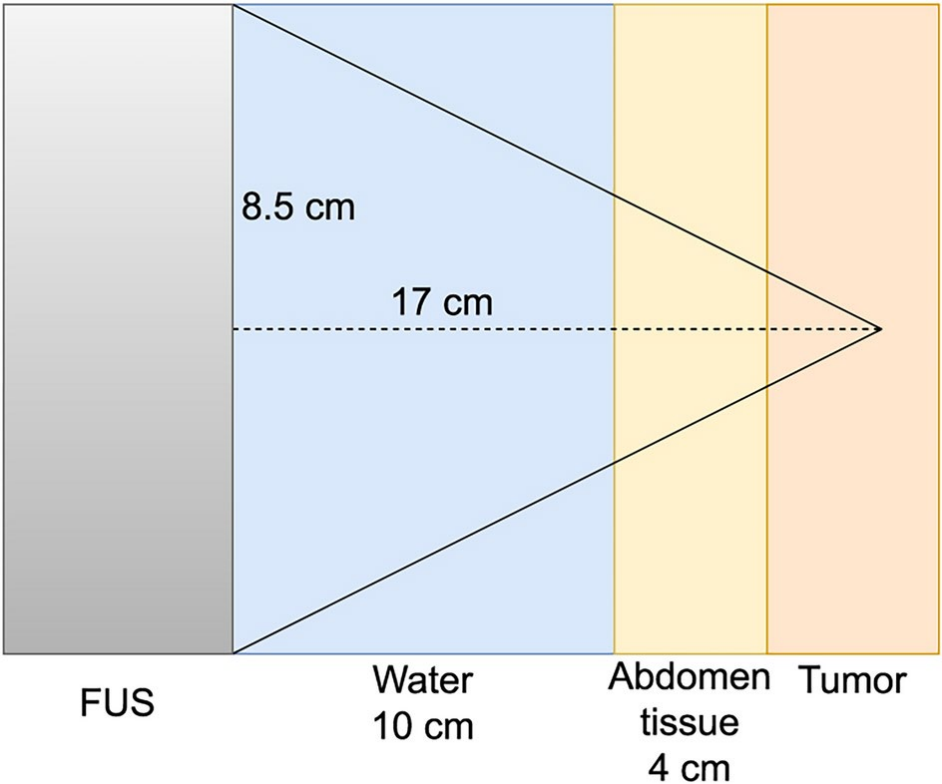
Schematic illustration of the abdomen base case. The tumor tissue is shown in orange, the yellow area shows the abdomen tissue and the blue area shows the water bath. The FUS probe is to the left-hand side indicated as the gray area. In the baseline simulation, the probe radius was ${8.5}\,\hbox {cm}$ and the focusing depth ${17.0}\,\hbox {cm}$.

We explored the treatment of a cancerous tumor in the abdomen using a system as illustrated in Fig. 2. The ultrasound probe was positioned ${10}\,\hbox {cm}$ away from the abdomen, with a water bath interposed between them. Within this hypothetical scenario, the tumor was located ${4}\,\hbox {cm}$ deep within the abdomen, separated from the water bath by tissue. In the base case simulation, the ultrasound probe operated at a frequency of $f={1}\,\hbox {MHz}$, with a radius of $a={8.5}\,\hbox {cm}$, a focusing diameter of $d={17}\,\hbox {cm}$, and exerted an acoustic power of $P={250}\,\hbox {W}.$ In addition, we also simulated using a probe radii of ${6.5}\,\hbox {cm}$ and ${9.5}\,\hbox {cm}$, frequency of ${0.5}\,\hbox {MHz}$ and power of ${125}\,\hbox {W}$ and ${500}\,\hbox {W}$.

### Numerical methods

We present the numerical procedures used. The ultrasound propagation pressure field was first calculated. Then, using the propagation pressure field, the temperature and the acoustic streaming were calculated.

Initially, we defined the configuration of the ultrasound probe and treatment area, see “Breast case” and “Abdomen case” sections. Subsequently, the designated area underwent appropriate discretization, utilizing the rotational symmetry perpendicular to the propagation axis. We computed next the propagation pressure field utilizing the WAKZK equation with 128 harmonics. For this computation, we utilized the updated 2019 version of the public domain high intensity therapeutic ultrasound (HITU) simulator^45^, which is a MATLAB code developed at the United States Food and Drug Administration. From the resultant pressure field, we obtained both the acoustic radiation force $\bar{{\mathbf{F}}}_2$ acting on the system and the absorbed heat *Q* using Eqs. (12) and (13).

In order to calculate the acoustic streaming phenomenon, we followed the methodology from our previous work^8^, utilizing Eq. (3). The analysis address solely cases that exhibit rotational symmetry around the propagation axis, allowing use of cylindrical coordinates. Equation (3) was discretized and solved numerically, with appropriate boundary conditions, allowing us to model the acoustic streaming behavior. We used homogeneous Neumann boundary condition at the propagation axis and homogeneous Dirichlet boundary conditions on all the other edges of the domain, i.e., $\bar{p}_2=0$.

The temperature rise within the medium was then computed employing Pennes’ bioheat Eq. (6), utilizing once again the updated 2019 version of the HITU simulator^45^, which we validated against experiments conducted by Huang et al.^16^, as well as our independently developed implementation, ensuring its accuracy. The rise in temperature was calculated for 15 seconds of treatment, which entails 15 FUS pulses of 1% DC. From the calculated rise in temperature we fitted the peak temperatures at each cycle to the Eq. (14) inspired by the Eq. (7) derived in the work of Bacon and Shaw^15^,

14 $$\begin{aligned} \Delta T(t)=l_1\ln (1+l_2(t+l_3)). \end{aligned}$$

Here $l_1$, $l_2$ and $l_3$ are fitted coefficients. A fitted version of Eq. (14) was used to calculate the rise in temperature for long treatment times, which would otherwise have been computationally expensive to calculate numerically.

## Results and discussion

In this section, we present and discuss the simulation results. We begin by examining, in detail, the temperature and streaming profiles for the breast and abdomen cases, with the baseline US parameters. Next, we examine the relationship between temperature and the time required to achieve a ${10}\,\upmu \hbox {m}$ streaming distance across various frequencies, power amplitudes, and FUS probe radii. Finally, we discuss the broader implications of our simulations for the cancer treatment field, drawing conclusions from our findings.

### Detailed analysis of breast and abdomen cases

**Fig. 3:**
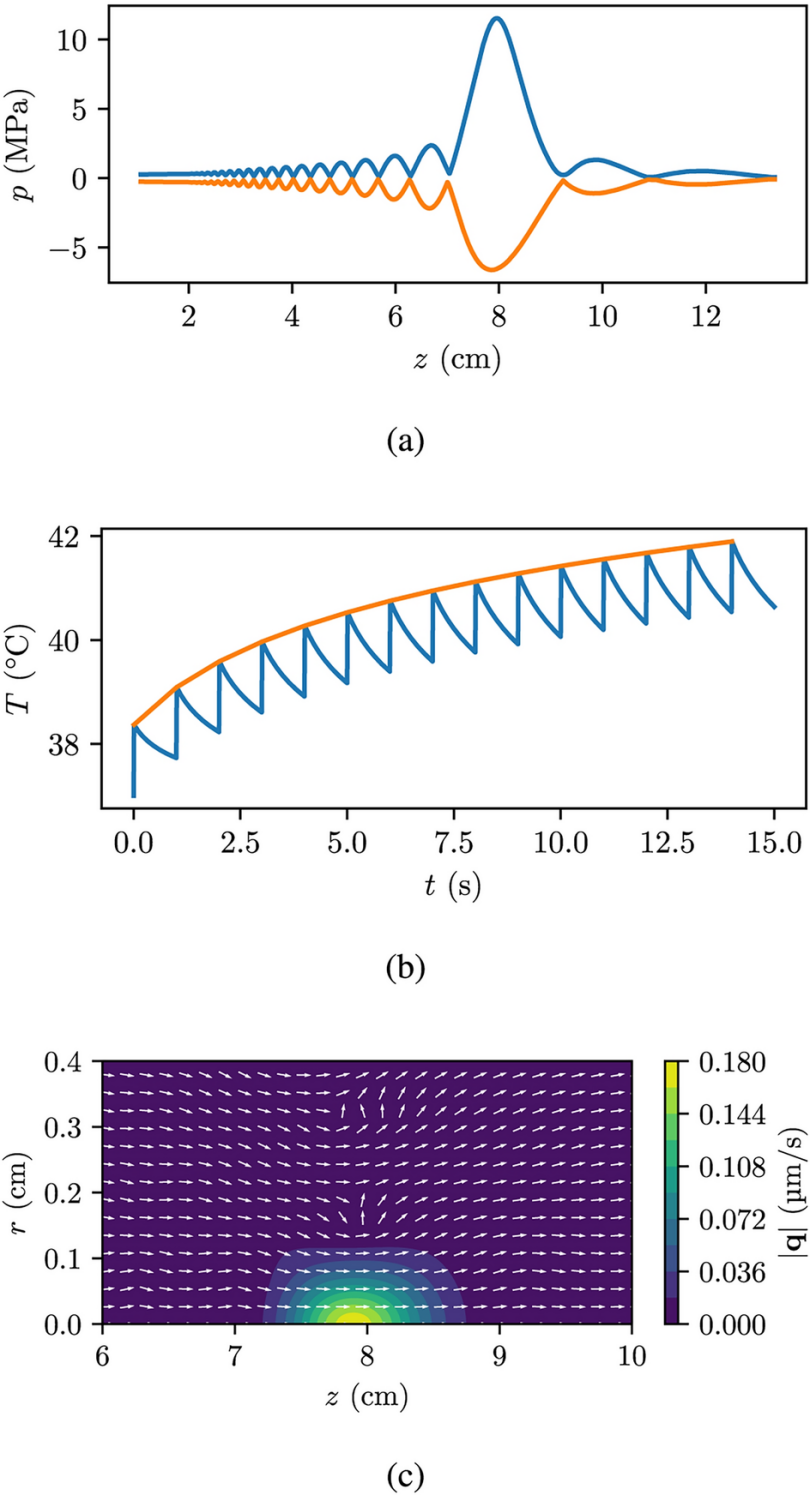
Simulation results from the breast base case as seen in Fig. 1. (**a**) Shows the peak compressional (blue) and rarefactional (orange) pressures along the axial axis. (**b**) Shows the maximum temperature (blue) and the fitted Eq. 14 (orange) as a function of time during a ${15}\,\hbox {s}$ period with a DC of 1% and a PRF of ${1}\,\hbox {Hz}.$ (**c**) shows the volumetric fluxes ${\mathbf{q}},$ where the white arrows show the direction of the volumetric flux and the contour indicates the magnitude.

**Fig. 4:**
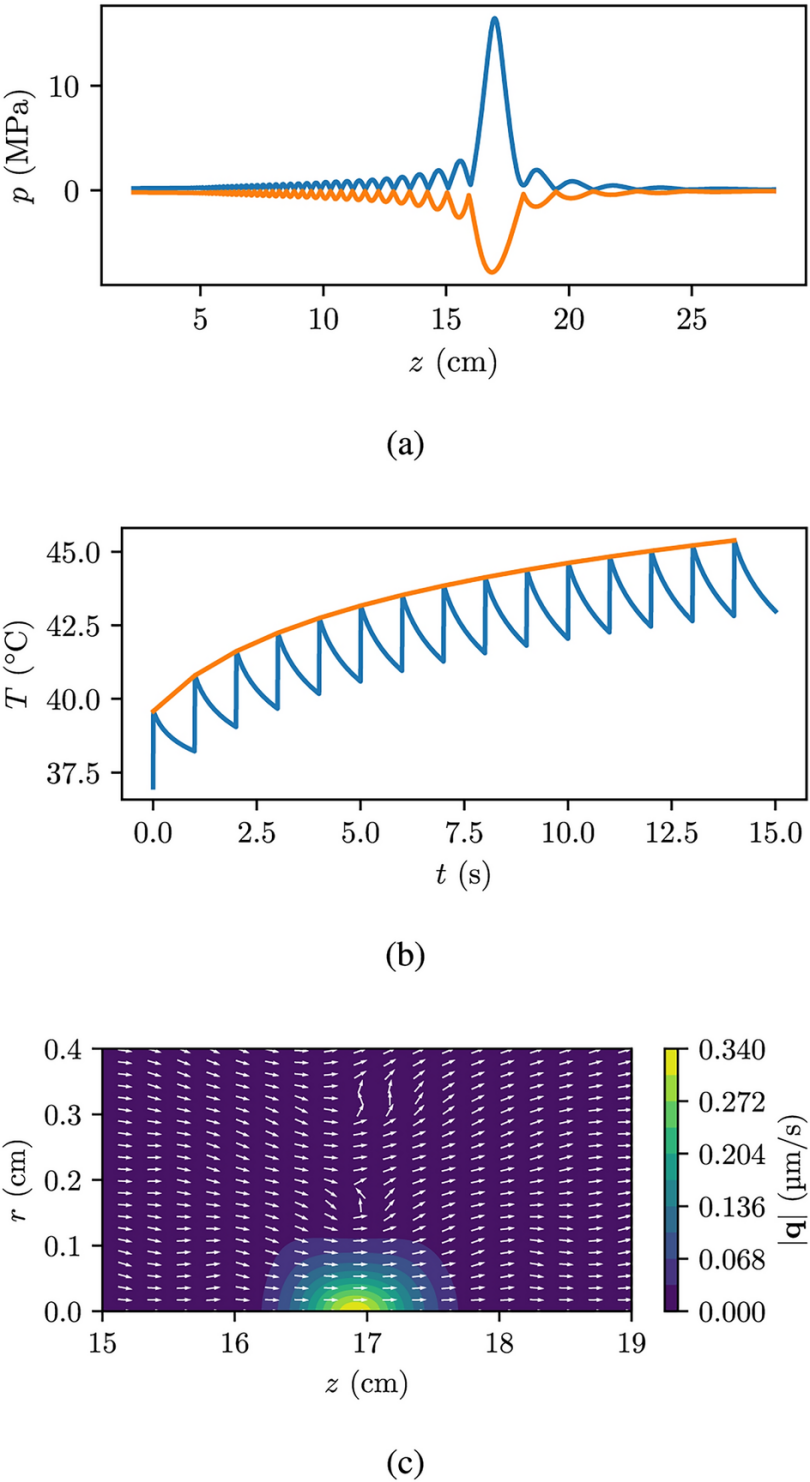
Simulation results from the abdomen base case as seen in Fig. 2. (**a**) Shows the peak compressional (blue) and rarefactional (orange) pressures along the axial axis. (**b**) Shows the maximum temperature (blue) and the fitted Eq. 14 (orange) as a function of time during a ${15}\,\hbox {s}$ period with a DC of 1% and a PRF of ${1}\,\hbox {Hz}.$ (**c**) shows the volumetric fluxes ${\mathbf{q}},$ where the white arrows show the direction of the volumetric flux and the contour indicates the magnitude.

Figure 3a shows the pressure along the propagation axis, the *z*-axis, at $r=0$ for the breast case, with the blue line representing the peak compressional pressure and the orange line representing the rarefactional pressure. Similarly, Fig. 4a shows the pressure for the abdomen case. Notably, both cases exhibits a significant nonlinear contribution.

Figures 3b and 4b show the maximum tumor temperature over time for the breast and abdomen cases, respectively, during FUS sonication with a PRF of ${1}\,\hbox {Hz}$ and a DC of 1%, as indicated by the blue line. The orange line in Figs. 3b and 4b represents the fitted Eq. 14. The fitting using Eq. 14 was fairly accurate having an average percentage deviation of less than 1 %, thus our decision to use this fitted function to calculate the temperature rise over extended periods of FUS exposure is reasonable. For the breast case, the temperature remains below the temperature threshold of ${50}^\circ \hbox {C}$ for the first ${15}\,\hbox {s}$. However, the temperature continues to increase over time, without reaching a steady-state value (see 14). This means that, despite the low DC, there is a limit to the time the treatment can go on before a longer break is necessary. This is particularly relevant for the abdomen case, which experiences a temperature increase of about ${12}^\circ \hbox {C}$ over a period of ${15}\,\hbox {s}$.

Lastly, Figs. 3c and 4c present the contour plots of the acoustic streaming amplitude for both the breast and abdomen cases, respectively. The order of magnitude in acoustic streaming is in micrometer per second. Since a 1% duty cycle is used, this streaming only acts to produce displacement 1% of the time and long treatments are therefore required to reach appreciable displacement distances.

### Effect of transducer parameters on temperature and treatment time

We will here discuss how the different transducer parameters affects the temperature and treatment time for both the breast and abdomen case.

**Fig. 5:**
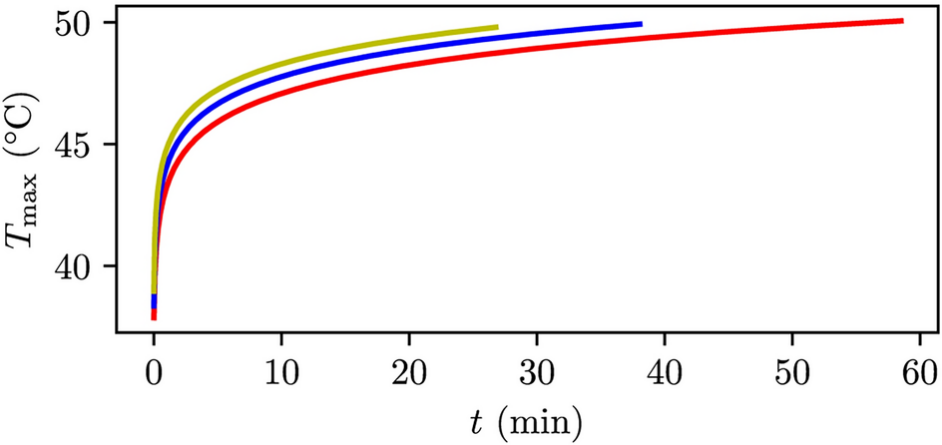
The maximum temperature $T_\text {max}$, as a function of time using the individually fitted Eq. 14 for the breast case with a FUS probe with frequency ${1}\,\hbox {MHz}$, power ${100}\,\hbox {W}$ and a probe radius of ${3.0}\,\hbox {cm}$ (red line), ${4.0}\,\hbox {cm}$ (blue line) and ${5.0}\,\hbox {cm}$ (yellow line). They have been plotted up to the point of having streamed ${10}\,\upmu \hbox {m}$ at focus.

Figure 5 illustrates the fitted Eq. 14 as a function of time for the breast case, using a FUS probe frequency of ${1}\,\hbox {MHz}$ and a power of $P={100}\,\hbox {W}$, for probe radii of ${3}\,\hbox {cm}$ (red line), ${4}\,\hbox {cm}$ (blue line) and ${5}\,\hbox {cm}$ (yellow line). These are plotted up to the point where the liquid has moved a distance of ${10}\,\upmu \hbox {m}$ due to acoustic streaming. The figure clearly demonstrates that a probe radius of ${5}\,\hbox {cm}$ achieves the shortest time to reach a distance of ${10}\,\upmu \hbox {m}$ while staying below the temperature threshold of ${50}^\circ \hbox {C}$. However, this radius also results in the smallest focus area, with a full width half maximum (FWHM) of ${1.05}\,\hbox {mm}$ in the radial direction and ${5.19}\,\hbox {mm}$ in the axial direction, compared to ${1.56}\,\hbox {mm}$ and ${13.94}\,\hbox {mm}$ for a probe radius of ${3}\,\hbox {cm}$. Thus, a smaller probe radius might actually be more beneficial, as it affects a larger area.

**Fig. 6:**
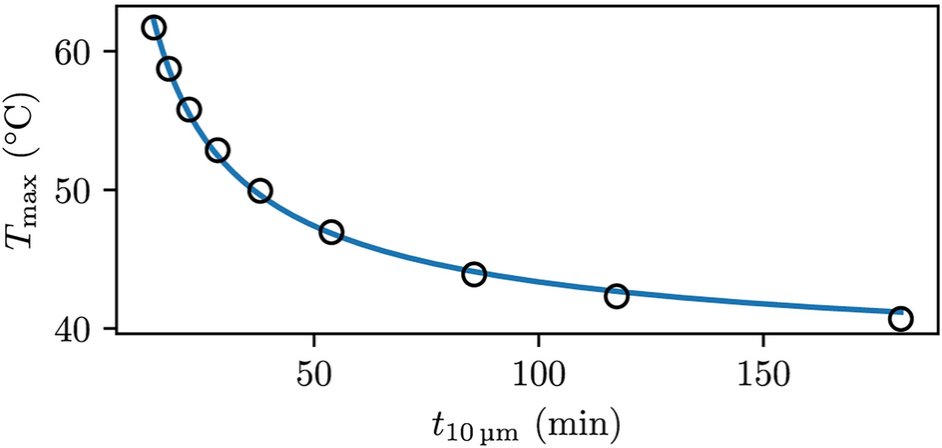
The maximum temperature using the individually fitted Eq. 14 against the time it takes to stream ${10}\,\upmu \hbox {m}$ denoted as $t_{{10}\,\upmu \hbox {m}}$, for the breast case using a FUS probe with frequency ${1}\,\hbox {MHz}$ and a probe radius ${4.0}\,\hbox {cm}$. Acoustic power values of 25.0, 37.5, 50.0, 75.0, 100.0, 125.0, 150.0, 175.0 and ${200.0}\,\hbox {W}$ were used, where the greater power value gives the higher temperature values shown as black circles. The fitted Eq. 15 is shown as the blue line.

In Fig. 6 the maximum temperature $T_\text {max}$, after the liquid has streamed ${10}\,\upmu \hbox {m}$ is plotted against the time required $t_{{10}\,\upmu \hbox {m}}$, using Eq. 5 for the breast base case, using different acoustic power values. We fitted the values to the function:

15 $$\begin{aligned} \Delta T(t)=L_1t^{-L_2}. \end{aligned}$$

where $L_1$ and $L_2$ are fitted coefficients with values 166.7 °C $\text{min}^{L_2}$ and 0.7 respectively and obtained a reasonable fit with an average percentage deviation of 3.6%. This shows that there is not a linear relationship between the time it takes for the liquid to flow ${10}\,\upmu \hbox {m}$ and the increase in temperature. When optimizing the FUS probe settings to shorten treatment time, it is important to recognize that beyond a certain point, any marginal reduction in treatment time leads to a substantial increase in temperature.

**Fig. 7:**
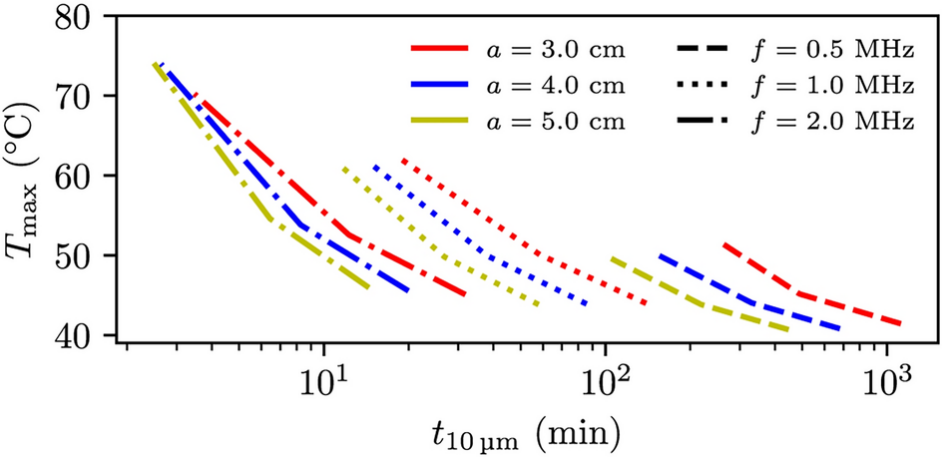
The maximum temperature using the individually fitted Eq. 14 against the time it takes for the fluid to stream ${10}\,\upmu \hbox {m}$ for the breast case. Acoustic power values of 50, 100 and ${200}\,\hbox {W}$ were used, where the greater power value gives the higher temperature. Dashed lines indicate a frequency of ${0.5}\,\hbox {MHz}$, dotted lines a frequency of ${1.0}\,\hbox {MHz}$ and dash-dotted lines a frequency of ${2.0}\,\hbox {MHz}$ with probe radius of ${3.0}\,\hbox {cm}$ (red), ${4.0}\,\hbox {cm}$ (blue) and ${5.0}\,\hbox {cm}$ (yellow).

**Fig. 8:**
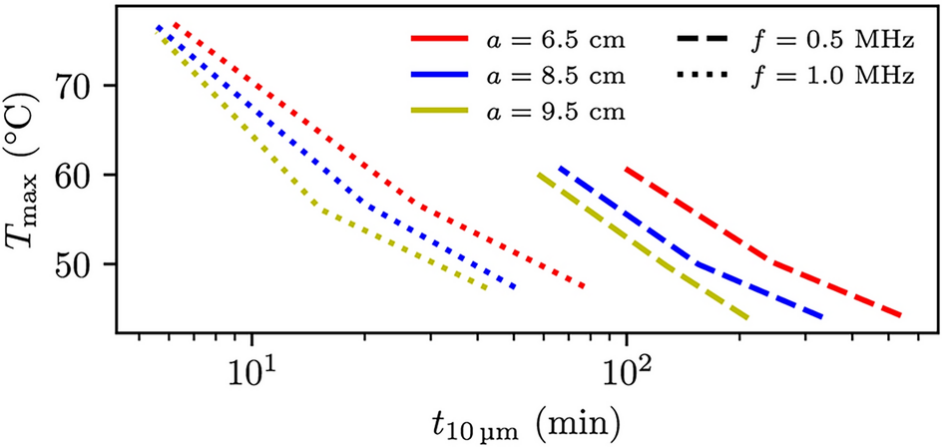
The maximum temperature using the individually fitted Eq. 14 against the time it takes for the fluid to stream ${10}\,\upmu \hbox {m}$ for the abdomen case. Acoustic power values of 125, 250 and ${500}\,\hbox {W}$ were used, where the greater power value gives the higher temperature. Dashed lines indicate a frequency of ${0.5}\,\hbox {MHz}$ and dotted lines a frequency of ${1.0}\,\hbox {MHz}$ with probe radius of ${6.5}\,\hbox {cm}$ (red), ${8.5}\,\hbox {cm}$ (blue) and ${9.5}\,\hbox {cm}$ (yellow).

Figure 7 shows the maximum temperature, (*y*-axis) after the liquid has streamed ${10}\,\upmu \hbox {m}$, plotted against the required time (*x*-axis) using Eq. 5 for different FUS probe settings for the breast case. The red line indicates a probe radius of $a={3}\,\hbox {cm}$, the blue line indicates a probe radius of $a={4}\,\hbox {cm}$ and the yellow line indicates a probe radius of $a={5}\,\hbox {cm}$. These probe radii correspond to F-numbers 1.3, 1.0 and 0.80 respectively. Dashed lines indicate a frequency of $f={0.5}\,\hbox {MHz}$, dotted lines indicate a frequency of $f={1.0}\,\hbox {MHz}$, while dash-dotted lines indicate a frequency of $f={2}\,\hbox {MHz}$. The lines illustrate the relationship between acoustic power and temperature increase, showing higher temperature and shorter required treatment time with greater acoustic power. This is due to the dependence of both acoustic streaming and heat dissipation on intensity, as indicated by Eqs. 1 and 13. The figure also indicates that a smaller probe radius results in a higher temperature, attributed to larger focal region. Additionally, a lower frequency results in a greater temperature relative to the time required to flow ${10}\,\upmu \hbox {m}$.

Figure 8 shows the maximum temperature (*y*-axis) after the liquid has streamed ${10}\,\upmu \hbox {m}$, plotted against the time taken (*x*-axis) using Eq. 5 for various FUS probe settings for the abdomen case. The red line represents a probe radius of $a={6.5}\,\hbox {cm}$, the blue line indicates a radius of $a={8.5}\,\hbox {cm}$, and the yellow line corresponds to a radius of $a={9.5}\,\hbox {cm}$. With the focusing depth remaining constant, these radii correspond to F-numbers of 1.3, 1.0, and 0.89, respectively. Dashed lines represent a frequency of $f={0.5}\,\hbox {MHz}$, while dotted lines represent $f={1.0}\,\hbox {MHz}$. The same relationships between the temperature and the transducer settings hold true for the abdomen case as for the breast case, though the temperature value is more pronounced for comparable acoustic streaming values. This suggests that the FUS temperature field in the breast case allows for more efficient heat transfer. Figure 7 indicates that the optimal FUS probe setting for minimizing treatment time, while staying below the temperature increase threshold of 50 °C, is a FUS probe with larger probe radius (more focused) and high frequency with the caveat that a smaller region is treated.

For longer treatment periods aiming to reach a distance of ${50}\,\upmu \hbox {m}$ while keeping the temperature below 50 °C, occasional breaks can be utilized to allow the temperature in the tumor to return to body temperature. In the breast case, it was possible to achieve a ${50}\,\upmu \hbox {m}$ distance in less than one and a half hour using a frequency of ${2}\,\hbox {MHz}$. Conversely, using a frequency of ${0.5}\,\hbox {MHz}$ could extend the treatment duration to up to 93 hours, highlighting the importance of selecting the appropriate FUS probe settings for effective patient treatment.

### Implications

To summarize, we have demonstrated the potential of acoustic streaming for two different tumor cases and estimated the time required to achieve specified streaming distances without excessively increasing the temperature.

The central part of a tumor often has poor vascularization and increased interstitial fluid pressure, thus diffusion is the mechanism for the transport of both NPs and free drugs not encapsulated in NPs. Diffusion is a very slow process for large particles and we have shown that acoustic streaming has the potential to facilitate transport.

We have shown that a higher frequency has the greatest positive effect in shortening the treatment time, while staying within a reasonable temperature. Increasing the probe radius has a smaller impact. The focusing area will in both instances decrease, which we recommend should be accounted for in future work as a smaller area will be treated. One method that could be potentially beneficial in this respect is beam steering, whereby the direction of the ultrasound beam is scanned either mechanically or electronically. This technique could allow clinicians to treat a larger area of the tumor by shifting the FUS focus and enabling one area of the tumor to cool while other areas are treated. This method is already employed in clinical settings for HIFU treatment^46,47^. There is also some development in robot-assisted HIFU treatment, in which the probe is moved mechanically by a robot^48^.

The hydraulic conductivity of 5 × 10^−13^ m^2^ Pa^−1^ s^−1^ used in this study represents a favorable scenario. There are, however, several studies that have reported hydraulic conductivity values on this order of magnitude^39–42^. Furthermore, a few studies suggest that pulsed FUS can enhance tumor tissue permeability by altering the tumor micro-environment and opening up intercellular spaces, thereby increasing fluid flow^49–51^. This supports the relevance of using a hydraulic conductivity in the upper end of the typical range for this study. Future work, however, should look into the possibility of treating tumors with lower hydraulic conductivity.

In all our simulations, we assumed axisymmetry and considered the breast, abdomen, and tumor tissues to be homogeneous, disregarding any heterogeneity. These breast and abdomen cases were chosen specifically so that heterogeneity due to bone could be excluded. In real scenarios, some heterogeneity is still expected and would especially influence hydraulic conductivity and perfusion. In this work, perfusion has been neglected and any effect of perfusion would be beneficial, as discussed below. Heterogeneity in the hydraulic conductivity is expected to influence the trends in Figs. 5, 6, 7 and 8 if variation occurs on the same length scale as the size of the focal spot.

Our calculations are conservative in that they assume the worst-case scenario for the temperature increase. Specifically, we assumed that all the heat generated by the acoustic dissipation must be transported away from the focal spot by thermal conduction, and we have neglected any potential cooling effect from perfusion. Depending on the tumor, the perfusion could have a significant cooling effect that limits temperature increase. This could, in turn, allow greater acoustic power and/or higher DC and thus an increased streaming effect. In future work, we recommend to study the effect that the highly variable perfusion might have, and to what extent it would allow for shorter treatment times and/or treatment of tumors with less favorable hydraulic conductivity values than has been assumed here.

## Conclusions

We set up two tumor cases: one for the breast and one for the abdomen. For both cases, we varied the focused ultrasound transducer radius, frequency, and power to simulate acoustic streaming and temperature rise. We fitted the simulated temperature rise to a logarithmic equation, allowing us to predict the rise in temperature for longer treatments and showing that despite a low duty cycle, the temperature does not reach a steady state.

Our simulations showed that higher frequencies and larger probe radii resulted in shorter treatment times relative to the temperature rise, at the cost of a smaller treated area. This pattern held for both cases. Additionally, there was a nonlinear relationship between the power used for the focused ultrasound probe and the rise in temperature.

Our results indicate that it may be possible to achieve reasonable acoustic streaming values in tumor without exceeding the temperature threshold. Treatment times for streaming 50 μm for the breast case ranged from less than one and a half hour to 93 hours, depending on focused ultrasound probe settings, highlighting the importance of selecting the proper settings.

We hope our simulations can guide the choice of focused ultrasound probe settings for treating tumors with ultrasound-induced drug delivery. Future work should include beam steering to explore treating larger areas using higher power on the focused ultrasound probe. Appropriate trade-offs involving the size of the treated area given the focusing of the beam, should also be compared. Furthermore, the present study has neglected the potentially beneficial effect of perfusion. We recommend to study the effect that perfusion might have, and to what extent it would allow for shorter treatment times and/or treatment of tumors with less favorable hydraulic conductivity values than has been assumed here.

## Data Availability

The datasets generated and/or analysed during the current study are available from the corresponding author on reasonable request.
